# Thyroid eye disease with teprotumumab-related hyperglycemia associated with increased growth hormone secretion

**DOI:** 10.1210/jcemcr/luag186

**Published:** 2026-07-13

**Authors:** Ken Sugawa, Yuji Hataya, Kimiaki Murabe, Kanta Fujimoto, Toshio Iwakura, Naoki Matsuoka

**Affiliations:** Department of Diabetes and Endocrinology, Kobe City Medical Center General Hospital, Kobe, Hyogo 650-0047, Japan; Department of Diabetes and Endocrinology, Kobe City Medical Center General Hospital, Kobe, Hyogo 650-0047, Japan; Department of Diabetes and Endocrinology, Kobe City Medical Center General Hospital, Kobe, Hyogo 650-0047, Japan; Department of Diabetes and Endocrinology, Kobe City Medical Center General Hospital, Kobe, Hyogo 650-0047, Japan; Department of Diabetes and Endocrinology, Kobe City Medical Center General Hospital, Kobe, Hyogo 650-0047, Japan; Department of Diabetes and Endocrinology, Kobe City Medical Center General Hospital, Kobe, Hyogo 650-0047, Japan

**Keywords:** teprotumumab, hyperglycemia, growth hormone, insulin-like growth factor 1, thyroid eye disease

## Abstract

Teprotumumab, an insulin-like growth factor-1 (IGF-1) receptor antibody, has been approved for thyroid eye disease (TED) treatment. However, teprotumumab is associated with adverse events such as hyperglycemia, and the underlying mechanisms of teprotumumab-related hyperglycemia remain unclear. We report a case of teprotumumab-related hyperglycemia with endocrinological evaluation. A 60-year-old woman with Graves disease developed active TED and was treated with teprotumumab. The patient had preexisting type 2 diabetes managed with oral hypoglycemic agents. At 6 weeks after teprotumumab initiation, glycemic control deteriorated, necessitating temporary teprotumumab discontinuation and insulin therapy initiation. Serum growth hormone (GH) level changed minimally, whereas serum IGF-1 level increased markedly, suggesting increased integrated GH secretion. Fasting plasma glucose level substantially increased, whereas serum immunoreactive insulin level only modestly increased, indicating insufficient endogenous insulin secretion. Following glycemic improvement with insulin therapy, teprotumumab was resumed, and 8 infusions were completed. Despite persistently elevated serum IGF-1 levels, insulin was gradually tapered with concomitant weight loss and was discontinued at 9 weeks after the final infusion. In patients with reduced endogenous insulin secretory capacity, teprotumumab-related hyperglycemia may develop when endogenous insulin secretion is insufficient to compensate for GH-mediated increases in hepatic gluconeogenesis and insulin resistance.

## Introduction

Thyroid eye disease (TED), an autoimmune disease characterized by inflammatory changes in orbital adipose tissues and extraocular muscles, is most commonly associated with Graves disease [1]. The pathogenesis of TED involves the activation of the thyroid-stimulating hormone receptor (TSHR) on orbital fibroblasts by TSHR antibodies. Additionally, the insulin-like growth factor-1 (IGF-1) receptor (IGF-1R), which is coexpressed with TSHR, forms a physical and functional complex that activates downstream signaling pathways promoting adipogenesis, myofibroblast differentiation, hyaluronan production, and inflammatory cytokine release.

Randomized controlled trials (RCTs) have demonstrated the efficacy of teprotumumab, a fully human monoclonal antibody targeting IGF-1R, in patients with moderate-to-severe active TED [2, 3]. However, teprotumumab therapy is associated with several adverse events such as hearing impairment, hyperglycemia, and infusion reactions [4]. Approximately 10% of patients in RCTs developed mild-to-moderate hyperglycemia, generally managed with antidiabetic therapy adjustment [5]. Recently, cases of severe hyperglycemia, including diabetic ketoacidosis and hyperosmolar hyperglycemic syndrome, have been reported [6-9]. Nevertheless, the underlying mechanisms remain poorly understood, and optimal management strategies have not been established.

Herein, we report a case of teprotumumab-related hyperglycemia with endocrinological evaluation after teprotumumab initiation.

## Case presentation

A 60-year-old woman diagnosed with Graves disease at the age of 59 years initially received thiamazole therapy. At 9 months after treatment initiation, the patient exhibited eyelid swelling and diplopia. While receiving thiamazole therapy at a dose of 15 mg/day, thyroid function tests indicated a thyroid-stimulating hormone level of 0.65 μIU/mL (SI: 0.65 mIU/L) (reference range, 0.61–4.23 μIU/mL [SI: 0.61-4.23 mIU/L]), free thyroxine level of 0.94 ng/dL (SI: 12.1 pmol/L) (reference range, 0.9-1.7 ng/dL [SI: 11.6-21.9 pmol/L]), and free triiodothyronine level of 3.53 pg/mL (SI: 5.43 pmol/L) (reference range, 2.3-4.0 pg/mL [SI: 3.5-6.1 pmol/L]). Thyroid autoantibody levels were elevated, with a thyrotropin receptor antibody level of 35.01 IU/L (reference range, 0-1.9 IU/L) and a thyroid-stimulating antibody level of ≥2000% (reference range, 0-179%).

The patient's medical history included dyslipidemia and gastric mucosa-associated lymphoid tissue lymphoma in remission. She was diagnosed with type 2 diabetes at the age of 46 years and treated with metformin (500 mg/day) and anagliptin (200 mg/day), with a hemoglobin A1c (HbA1c) level of approximately 7.0% (SI: 53.0 mmol/mol) (reference range, 4.6-6.2% [SI: 26.8-44.3 mmol/mol]). The patient had no history of smoking and was deemed overweight (height: 157.0 cm, weight: 69.5 kg, body mass index: 28.2 kg/m^2^).

## Diagnostic assessment

Ophthalmologic examination revealed proptosis measuring 18 mm in both eyes, as well as diplopia in secondary gaze. A Clinical Activity Score of 5 was assigned based on the presence of spontaneous retrobulbar pain, conjunctival redness, and swelling of the caruncle, eyelids, and conjunctiva [10]. Orbital magnetic resonance imaging (MRI) showed extraocular muscle enlargement with high signal intensity on short tau inversion recovery (STIR) sequences, confirming active TED (Fig. 1).

**Figure 1:**
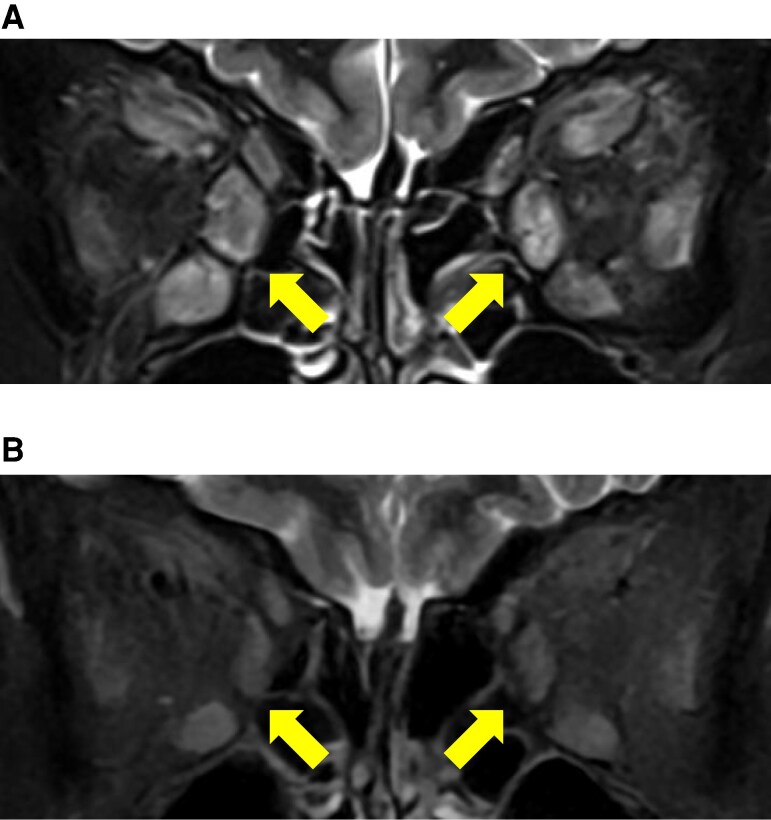
Orbital magnetic resonance imaging. (A) Prior to teprotumumab initiation, extraocular muscle enlargement with high signal intensity on short tau inversion recovery (STIR) sequences was observed (arrows), consistent with active thyroid eye disease. (B) Following the completion of teprotumumab therapy, extraocular muscle enlargement improved and STIR signal intensity decreased (arrows), indicating reduced disease activity.

## Treatment

After consulting with the ophthalmology department, teprotumumab therapy was initiated. At 3 weeks prior to the first infusion (baseline), the fasting plasma glucose (FPG) level was 135 mg/dL (SI: 7.5 mmol/L) (reference range, 70-110 mg/dL [SI: 3.9-6.1 mmol/L]), whereas the HbA1c level was 7.2% (SI: 55.2 mmol/mol) (Fig. 2). At 3 weeks after teprotumumab initiation, the FPG level increased to 229 mg/dL (SI: 12.7 mmol/L) before the second infusion; however, the second infusion was administered as scheduled because the patient remained asymptomatic. At 6 weeks after teprotumumab initiation, the FPG and HbA1c levels further increased to 312 mg/dL (SI: 17.3 mmol/L) and 10.5% (SI: 91.3 mmol/mol), respectively, with negative urine ketones. Consequently, teprotumumab therapy was temporarily discontinued.

**Figure 2:**
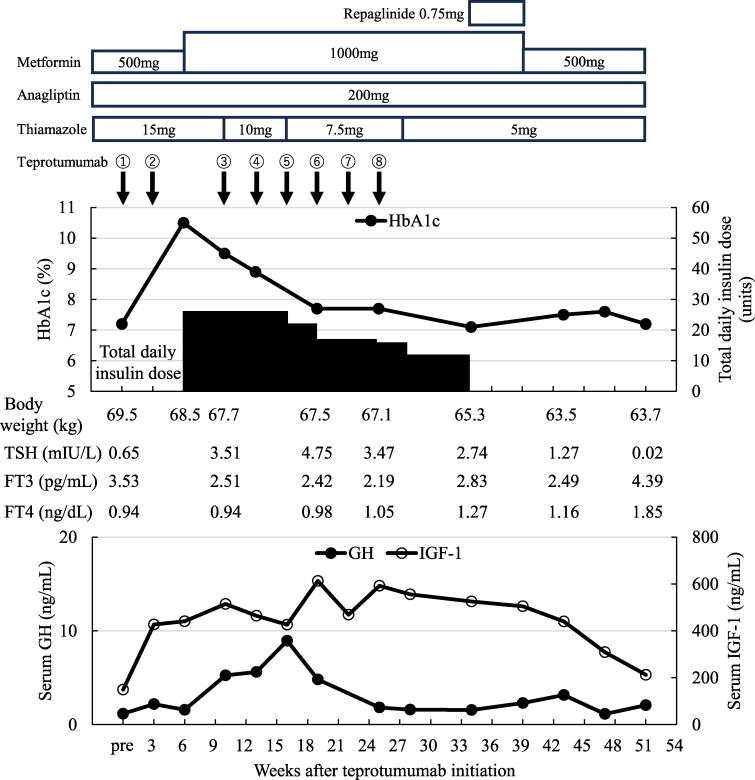
Clinical course after teprotumumab initiation. Glycemic control deteriorated rapidly at 6 weeks, prompting temporary discontinuation of teprotumumab and initiation of insulin therapy. After resumption, the total daily insulin dose was gradually reduced, and insulin was discontinued at 9 weeks after the final infusion (week 34). Body weight progressively decreased, while thyroid function remained stable under thiamazole therapy, as assessed by thyroid-stimulating hormone (TSH), free triiodothyronine (FT3), and free thyroxine (FT4). Serum growth hormone (GH) level remained within the normal range. Serum insulin-like growth factor-1 (IGF-1) level increased markedly and remained persistently elevated until 18 weeks after the final infusion (week 43); at this point, it began to decline. “Pre” indicates 3 weeks before teprotumumab initiation.

To improve glycemic control, the metformin dose was increased to 1000 mg/day, and intensive insulin therapy with continuous glucose monitoring was initiated at a total daily dose of 26 units. One month later, the FPG and HbA1c levels decreased to 130 mg/dL (SI: 7.2 mmol/L) and 9.5% (SI: 80.3 mmol/mol), respectively. With marked improvement of ocular symptoms, teprotumumab therapy was resumed at the patient's request, and a total of 8 infusions were completed. The total daily insulin dose was gradually reduced to 16 units by week 25, when the eighth and final infusion was administered. Insulin therapy was subsequently discontinued 9 weeks later (week 34). The Clinical Activity Score improved from 5 to 1, with only residual eyelid swelling, and orbital MRI showed improvement in extraocular muscle enlargement and STIR hyperintensity (Fig. 1). Body weight decreased from 69.5 kg at baseline to 67.1 kg at the final infusion (week 25) and to 63.5 kg 18 weeks later (week 43). Thyroid function remained stable on thiamazole therapy throughout the clinical course.

## Outcome and follow-up

Endocrinological evaluation was conducted to investigate the underlying mechanisms of hyperglycemia. The serum growth hormone (GH) level changed minimally from 1.15 ng/mL (SI: 1.15 μg/L) (reference range, 0.13-9.88 ng/mL [SI: 0.13-9.88 μg/L]) at baseline to 2.18 ng/mL (SI: 2.18 μg/L) at 3 weeks after teprotumumab initiation and remained within the normal range throughout the clinical course (Fig. 2). In contrast, the serum IGF-1 level increased markedly from 149 ng/mL (SI: 19.5 nmol/L; +0.8 SD) (age-adjusted reference range, 70-201 ng/mL [SI: 9.1-26.2 nmol/L]) at baseline to 427 ng/mL (SI: 55.5 nmol/L; +5.5 SD) at 3 weeks after teprotumumab initiation, suggesting increased integrated GH secretion. The serum IGF-1 level continued to rise during teprotumumab therapy and reached 592 ng/mL (SI: 77.0 nmol/L; +6.9 SD) by the time of the eighth and final infusion (week 25). It remained persistently elevated until 18 weeks after the final infusion (week 43), thereafter declining gradually and reaching 212 ng/mL (SI: 27.7 nmol/L; +2.3 SD) at 27 weeks after the final infusion (week 52).

At baseline, the FPG level was 135 mg/dL (SI: 7.5 mmol/L), and the serum immunoreactive insulin (IRI) level was 13.7 μU/mL (SI: 94.9 pmol/L) (reference range, 5-15 μU/mL [SI: 34.6-103.8 pmol/L]) (Fig. 3). The homeostasis model assessment for insulin resistance (HOMA-IR) was 4.57, and that for β-cell function (HOMA-β) was 68.5%, indicating insulin resistance (HOMA-IR >2.5) and relatively insufficient compensatory endogenous insulin secretion [11]. At 3 weeks after teprotumumab initiation, the FPG level increased to 229 mg/dL (SI: 12.7 mmol/L), whereas the serum IRI level modestly increased to 17.9 μU/mL (SI: 124.3 pmol/L), indicating an insufficient endogenous insulin secretory response to hyperglycemia. At 27 weeks after the final infusion (week 52), the FPG level was 126 mg/dL (SI: 7.0 mmol/L), and the serum IRI level was 9.6 μU/mL (SI: 66.5 pmol/L), with a HOMA-IR of 3.0, indicating improvement in insulin resistance.

**Figure 3:**
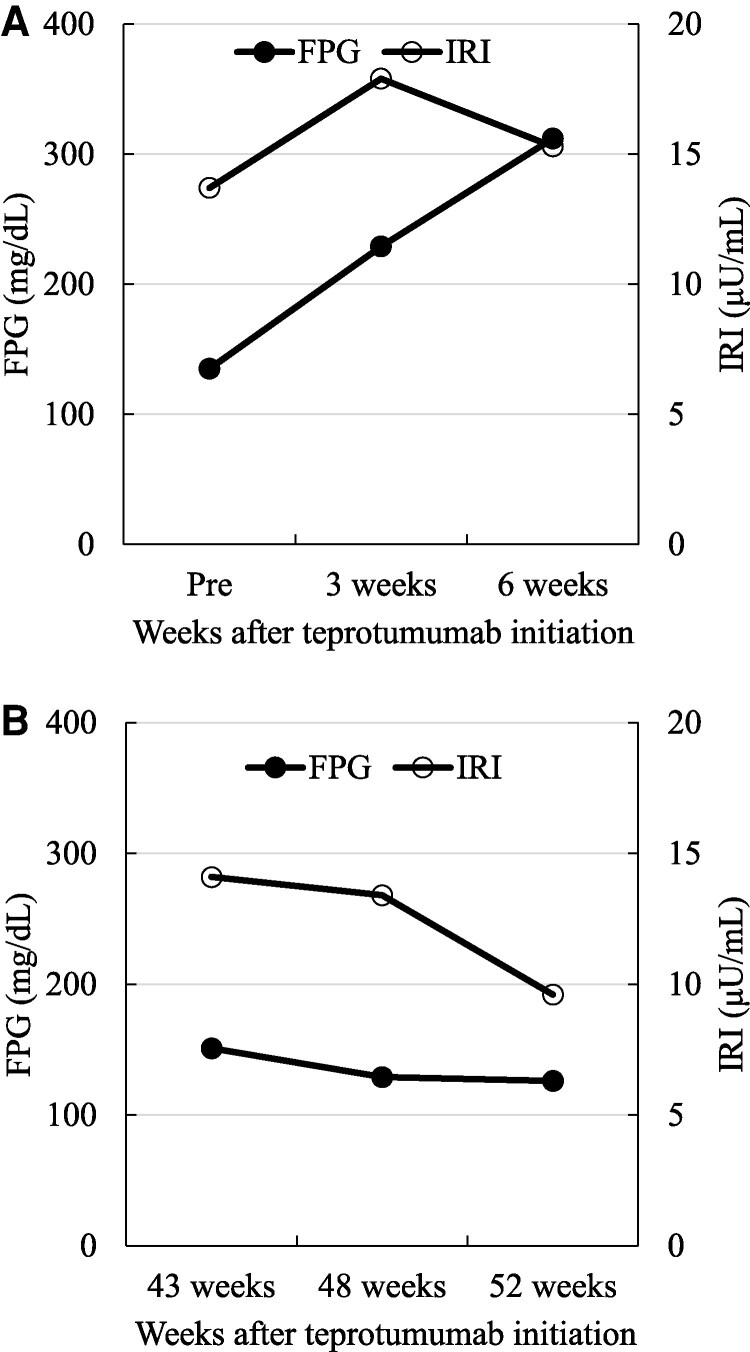
Changes in fasting plasma glucose (FPG) and immunoreactive insulin (IRI) levels. (A) FPG and IRI levels at baseline (Pre) and at 3 and 6 weeks after teprotumumab initiation. (B) FPG and IRI levels at 43, 48, and 52 weeks after teprotumumab initiation (18, 23, and 27 weeks after the final infusion). “Pre” indicates 3 weeks before teprotumumab initiation.

## Discussion

The present case involved severe hyperglycemia, accompanied by a marked increase in serum IGF-1 level despite minimal change in serum GH level. Because GH is secreted in a pulsatile manner, a single random measurement cannot reliably reflect overall GH secretory status; by contrast, IGF-1 serves as a stable surrogate marker of integrated 24-hour GH secretion [12]. Indeed, cases of acromegaly with apparently normal random GH levels but elevated IGF-1 levels have been reported [13]. This IGF-1 elevation may be explained by IGF-1R antibody-mediated attenuation of pituitary negative feedback, with the resulting GH excess enhancing hepatic IGF-1 production [14]. In addition, IGF-1R antibody may also contribute to IGF-1 elevation by reducing receptor-mediated clearance of IGF-1 through blockade of IGF-1 binding to IGF-1R [15]. A phase II trial of R1507, an IGF-1R antibody developed as a precursor to teprotumumab, showed a peak serum IGF-1 increase of approximately 300% from baseline in patients with sarcoma [16], comparable to that observed in the present case. Notably, a substantial rise in serum IGF-1 level was already apparent at 3 weeks, coinciding with the rapid deterioration of glycemic control. This observation supports a contribution of increased GH secretion to teprotumumab-related hyperglycemia. In contrast, an alternative mechanism involving teprotumumab-mediated direct interference with insulin receptor signaling through IGF-1R/insulin receptor hybrid receptors has also been proposed. However, its contribution to glucose metabolism is likely limited given that these receptors are preferentially activated by IGF-1 rather than insulin [1].

Before teprotumumab initiation, our patient already had insulin resistance and reduced endogenous insulin secretory capacity. After teprotumumab initiation, serum IRI rose only modestly despite severe hyperglycemia, and glycemic control improved rapidly with a relatively low insulin dose. Although endogenous insulin secretion and insulin resistance during teprotumumab treatment could not be precisely evaluated, these observations suggest insufficient compensatory insulin secretion in response to the hyperglycemia. Reported risk factors for teprotumumab-related hyperglycemia—older age, preexisting diabetes, and Asian or Hispanic ethnicity [17]—may have a common feature of relatively reduced endogenous insulin secretory capacity. To our knowledge, this is the first report to document serial changes in GH, IGF-1, and IRI after teprotumumab initiation. Our findings suggest that teprotumumab-related hyperglycemia may involve a failure of endogenous insulin secretion to compensate for GH-mediated increases in hepatic gluconeogenesis and insulin resistance.

A previous review reported body weight loss exceeding 5 kg in 7% to 12% of teprotumumab-treated patients in RCTs [18]; however, the underlying mechanism remains unclear. In the present case, body weight decreased by 6.0 kg. Thyroid function remained stable throughout, and weight loss continued even after glycemic improvement with insulin therapy, suggesting that neither thyrotoxicosis nor hyperglycemia was the primary cause. However, a voluntary reduction in dietary intake in response to hyperglycemia cannot be ruled out. The gradual reduction in the total daily insulin dose despite persistently elevated serum IGF-1 levels may reflect a contribution of improved insulin sensitivity associated with weight loss. Previous studies showed that blood glucose tended to increase early after teprotumumab initiation and subsequently stabilized [5, 17]. However, these studies did not demonstrate the relationship between body weight changes and glycemic variability. Further studies are needed to clarify the mechanism of teprotumumab-related weight loss and its effect on glucose metabolism.

In conclusion, teprotumumab-related hyperglycemia may develop when insulin secretion is insufficient to compensate for GH-mediated increases in hepatic gluconeogenesis and insulin resistance, particularly in patients with reduced endogenous insulin secretory capacity. Further studies are warranted to elucidate the underlying pathophysiology and develop optimal management strategies.

## Learning points

- Teprotumumab therapy may lead to rapid and severe hyperglycemia, sometimes necessitating temporary teprotumumab discontinuation and insulin therapy initiation.
- Teprotumumab-related hyperglycemia may develop when insulin secretion is insufficient to compensate for GH-mediated increases in hepatic gluconeogenesis and insulin resistance, particularly in patients with reduced endogenous insulin secretory capacity.
- Weight loss after teprotumumab initiation may contribute to subsequent glycemic control stabilization.

## Data Availability

The original data generated and analyzed during this study are included in the published article.
